# Supplementary material for: Right hemispheric white matter hyperintensities improve the prediction of spatial neglect severity in acute stroke
Source: Neuroimage Clin. 2022 Nov 11;36:103265. doi: 10.1016/j.nicl.2022.103265 (PMC9723300; doi:10.1016/j.nicl.2022.103265)
Supplement: Supplementary data 1 [file mmc1.pdf]

Supplementary Material

Right hemispheric white matter hyperintensities improve the prediction of spatial neglect severity in acute stroke

Lisa Röhrig[a], Christoph Sperber[a], Leonardo Bonilha[b], Christopher Rorden[c] and Hans-Otto Karnath[a,c]

Author affiliations:

[a] Division of Neuropsychology, Center of Neurology, Hertie-Institute for Clinical Brain Research, University of Tübingen, 72076 Tübingen, Germany

[b] Department of Neurology, Emory University, Atlanta, GA 30322, USA

[c] Department of Psychology, University of South Carolina, Columbia, SC 29208, USA

Correspondence to: Hans-Otto-Karnath, MD, PhD
Center of Neurology, University of Tübingen, Hoppe-Seyler-Str. 3, 72076 Tübingen, Germany
karnath@uni-tuebingen.de

Table S1. Correlations between patients' clinical and demographic parameters

						**CoC**
					**Age**	0.16
				**DS-WMH**	0.41	0.03
			**PV-WMH**	0.36	0.39	-0.02
		**Fazekas**	0.74	0.87	0.48	0.01
	**CHS**	0.86	0.67	0.74	0.53	-0.03
**Volume**	0.89	0.84	0.61	0.75	0.45	-0.08

Correlations were calculated between the different measures of white matter hyperintensities (WMH), age, and severity of spatial neglect (CoC). Correlation coefficients according to Spearman (r_s), if an ordinal variable was involved, or Pearson (r_p) are presented. All correlations were highly significant ($p < 0.001$), except for the correlations involving the CoC score ($p > 0.05$).

Abbreviations: *Volume* – normalized bilateral WMH volume (in cm^3), *CHS* – Cardiovascular Health Study scale value, *Fazekas* – overall score of Fazekas scale (PV-WMH and DS-WMH scores summed up), *PV-WMH* – Fazekas periventricular WMH score, *DS-WMH* – Fazekas deep subcortical WMH score, *CoC* – Center of Cancellation.

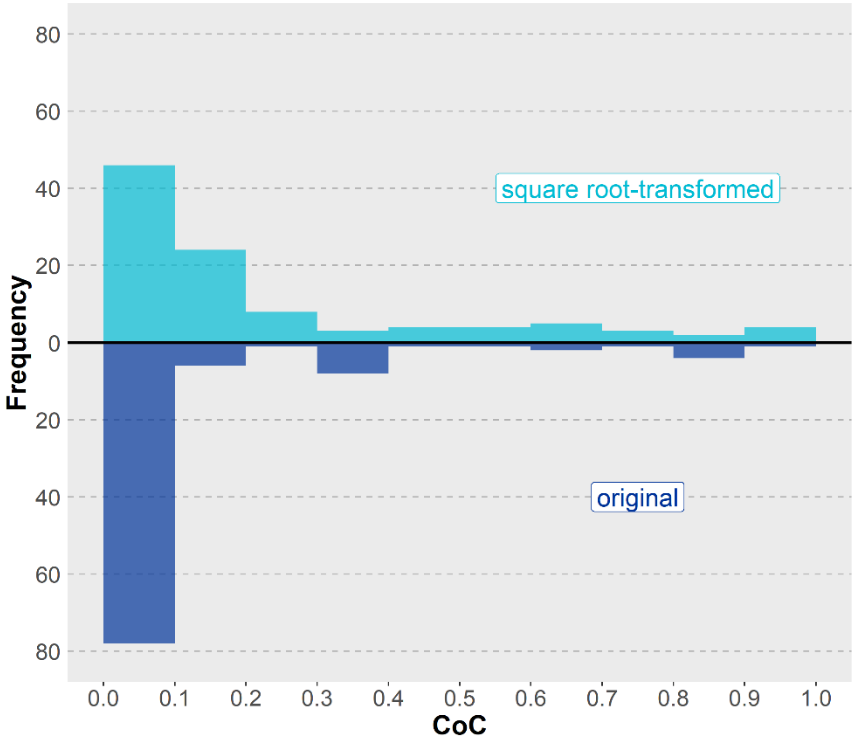

Figure S1. Behavioral outcome. The distributions of the original behavioral outcome (Center of Cancellation, CoC; dark blue) and the square root-transformed outcome (cyan blue) of all patients (*N* = 103) are visualized in a mirrored histogram. The square root-transformed CoC scores were distributed less skewed and were therefore used in further analyses. Note that negative (non-pathological) CoC scores were already eliminated by setting them to zero (*N* = 13 with a *mean original CoC* = -0.006).

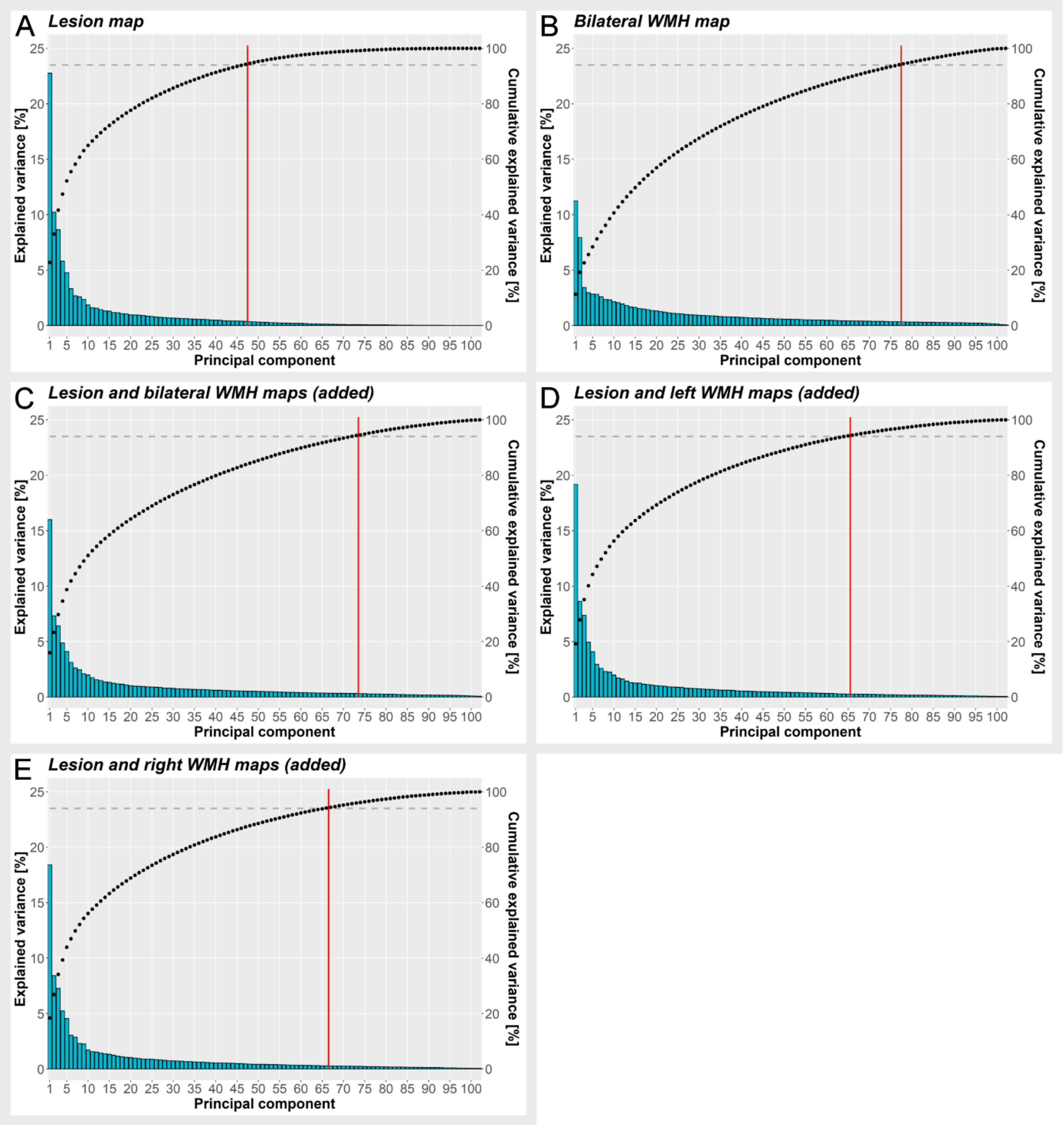

Figure S2. Principal components and explained variance. Results are shown that were obtained by principal component analyses (PCA) of voxel-wise maps of stroke lesion and/or white matter hyperintensities (WMH). Cyan blue-colored bars present the relative explained variance for each component (left y-axis). Black-colored dots visualize the relative cumulative explained variance (right y-axis). The grey-colored dashed horizontal line represents 94% cumulative explained variance, which was obtained by *N* principal components depicted by the red-colored vertical line (**A** – 47, **B** – 77, **C** – 73, **D** – 65, **E** – 66, respectively). Those *N* components were used for the prediction analyses; in case of concatenation of lesion and bilateral WMH, the components of lesion map (**A**) and WMH map (**B**) were concatenated (i.e., total *N* of 124).

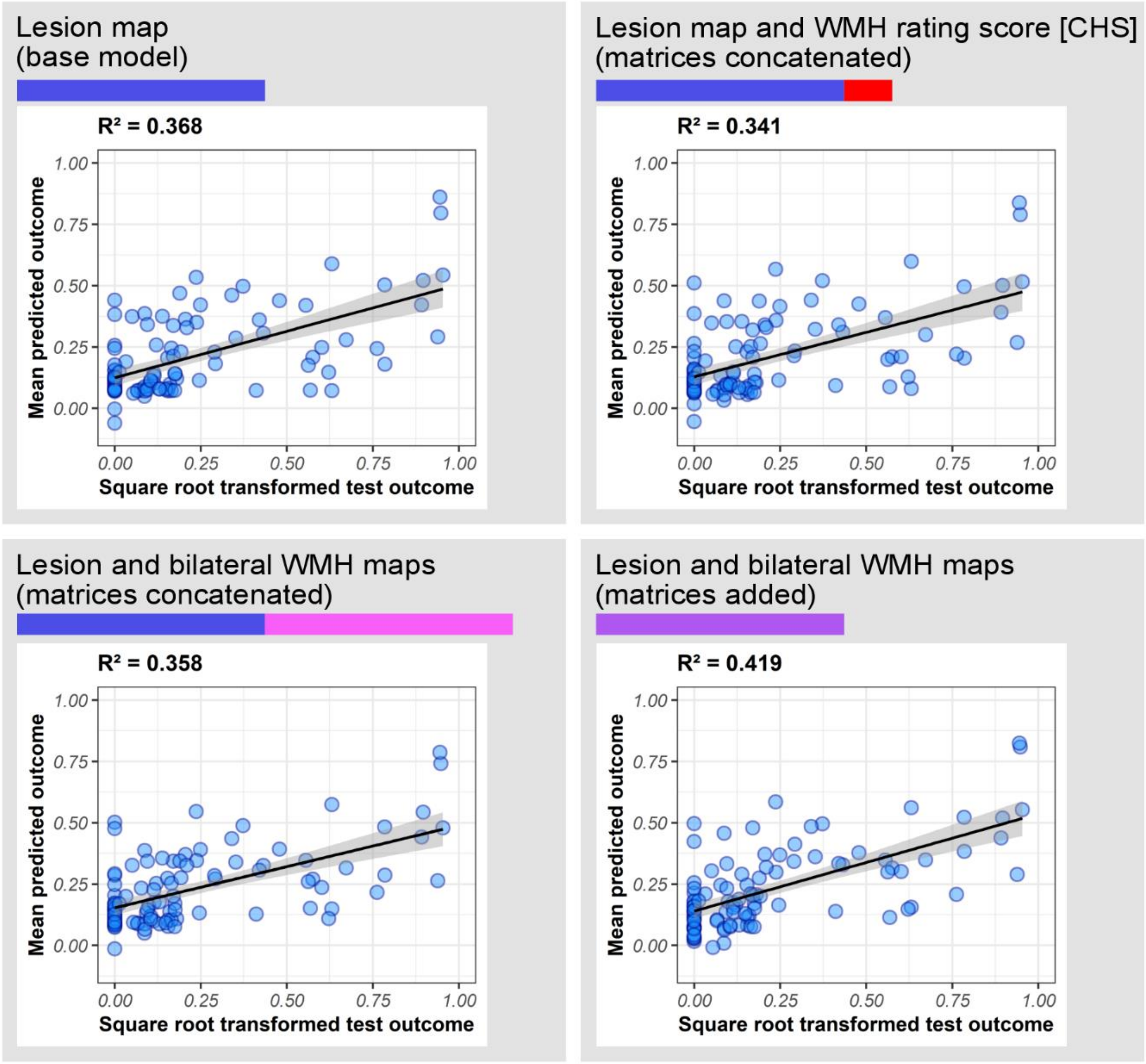

Figure S3. Model fits across model versions. Performances of the cross-validated and averaged models are displayed for prediction of neglect severity (CoC score). The different feature matrices are visualized as colored bars as in Figure 1B (main article). *Coefficient of Determination* (R^2) is reported as prediction accuracy. Scatter plots show the relationship of the test outcomes and the predictions averaged across 10 model repetitions; a linear model line is depicted with its regarding 95%-confidence interval (grey).

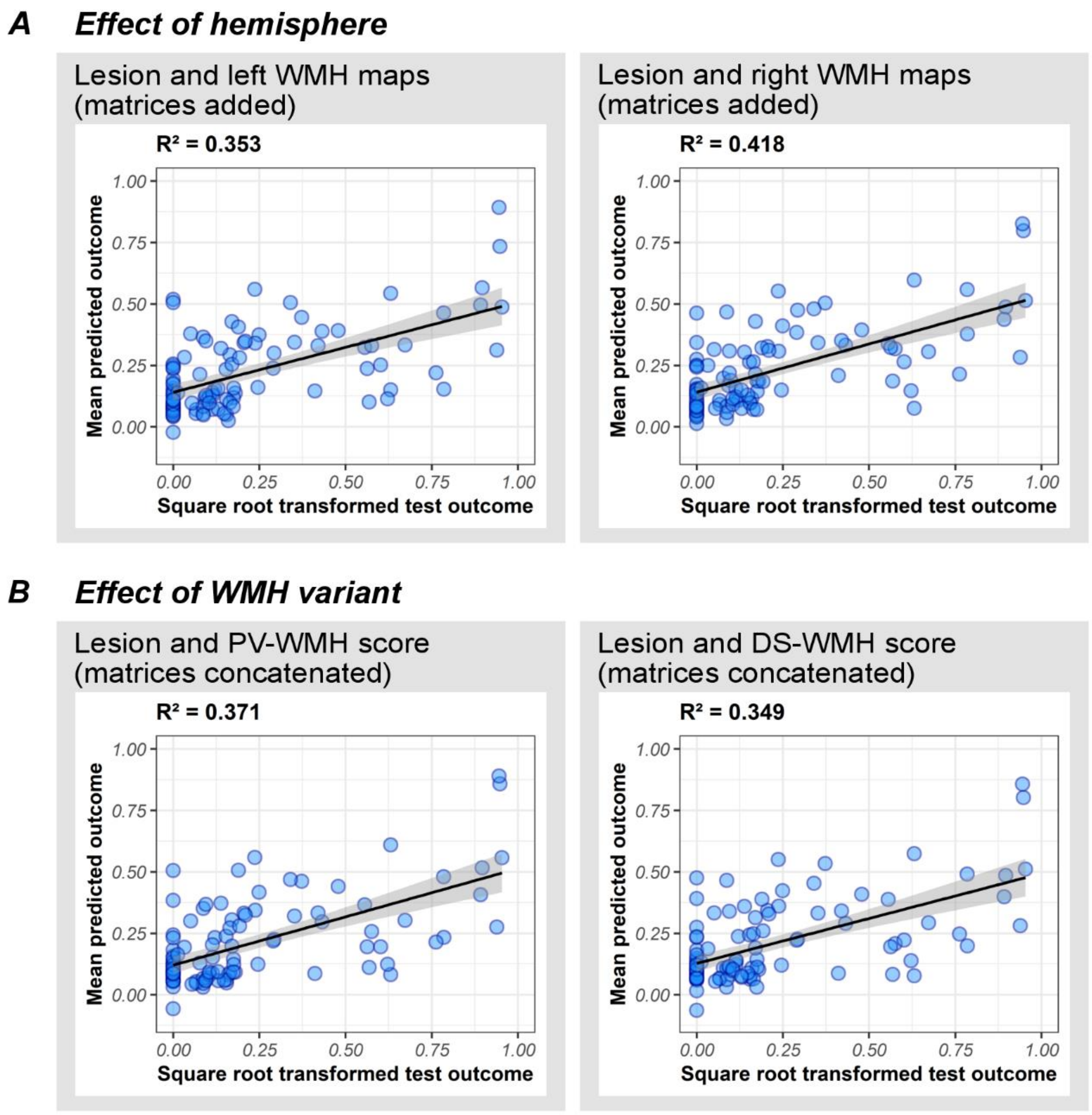

Figure S4. Effect of WMH localization on model fits. Illustrated are the effects of (**A**) hemisphere (left versus right hemispheric WMH) and (**B**) WMH variant (periventricular WMH [PV-WMH] versus deep subcortical WMH [DS-WMH]) on the model fits. For the effect of hemisphere, lesion and unilateral WMH maps were added. For the effect of WMH variant, lesion map and Fazekas rating scores were concatenated. Performances of the cross-validated and averaged models are displayed for the prediction of neglect severity (CoC score). *Coefficient of Determination* (R^2) is reported as prediction accuracy. Scatter plots show the relationship of the test outcomes and the predictions averaged across 10 model repetitions; a linear model line is depicted with its regarding 95%-confidence interval (grey).
